# Vascular Multiplicity Should Not Be a Contra-Indication for Live Kidney Donation and Transplantation

**DOI:** 10.1371/journal.pone.0153460

**Published:** 2016-04-14

**Authors:** Jeffrey A. Lafranca, Mark van Bruggen, Hendrikus J. A. N. Kimenai, Thi C. K. Tran, Türkan Terkivatan, Michiel G. H. Betjes, Jan N. M. IJzermans, Frank J. M. F. Dor

**Affiliations:** 1 Department of Surgery, division of HPB & Transplant Surgery, Erasmus MC, University Medical Center Rotterdam, Rotterdam, The Netherlands; 2 Department of Internal Medicine, Erasmus MC, University Medical Center Rotterdam, Rotterdam, The Netherlands; UNIFESP Federal University of São Paulo, BRAZIL

## Abstract

**Background:**

Whether vascular multiplicity should be considered as contraindication and therefore ‘extended donor criterion’ is still under debate.

**Methods:**

Data from all live kidney donors from 2006–2013 (n = 951) was retrospectively reviewed. Vascular anatomy as imaged by MRA, CTA or other modalities was compared with intraoperative findings. Furthermore, the influence of vascular multiplicity on outcome of donors and recipients was studied.

**Results:**

In 237 out of 951 donors (25%), vascular multiplicity was present. CTA had the highest accuracy levels regarding vascular anatomy assessment. Regarding outcome of donors with vascular multiplicity, warm ischemia time (WIT) and skin-to-skin time were significantly longer if arterial multiplicity (AM) was present (5.1 vs. 4.0 mins and 202 vs. 178 mins). Skin-to-skin time was significantly longer, and complication rates were higher in donors with venous multiplicity (203 vs. 180 mins and 17.2% vs. 8.4%). Outcome of renal transplant recipients showed a significantly increased WIT (30 vs. 26.7 minutes), higher rate of DGF (13.9% vs. 6.9%) and lower rate of BPAR (6.9% vs. 13.9%) in patients receiving a kidney with AM compared to kidneys with singular anatomy.

**Conclusions:**

We conclude that vascular multiplicity should not be a contra-indication, since it has little impact on clinical outcome in the donor as well as in renal transplant recipients.

## Introduction

Live kidney donation has become increasingly important over the years in the field of kidney transplantation (KT) and accounts for more than 55–60% of KT in The Netherlands [1]. Careful donor selection is essential to ensure donor safety. Due to the persistent donor organ shortage and increasing incidence of end-stage renal disease (ESRD) [2], there is a worldwide trend in accepting so called extended criteria live kidney donors (i.e. obese donors, older donors, donors with hypertension) [3]. Vascular multiplicity in live kidney donors is considered another extended criterion, because of the premise that it is associated with higher (surgical) complication rates in the recipient [4–6]. However, kidneys with multiple arteries and/or veins are common in kidney donors (between 18% and 25,1%) [7–9], and because of the donor shortage, it is likely that the acceptance of these kidneys for donation and transplantation will further increase over the years. Available literature suggest that outcome in donors with vascular multiplicity is excellent [10,11]. In 2008, Kok et al. investigated the live kidney donor cohort in our center from 2001 until 2005 regarding vascular multiplicity, showing that despite an increased warm ischemia time, operation time and increased blood loss in donors with multiple renal arteries, AM does not seem to be a contra-indication for donation [12]. They reported, however, an increased incidence of urological complications after KT in donors with AM. During the timespan of the analysed cohort, predominantly MRA and DSA were used as screening modalities for live kidney donors. Therefore, venous anatomy was not included in analyses, because renal veins are not visible with DSA. Since that time, our live kidney donation program has increased significantly, to the largest program in Europe with regular tertiary referrals, resulting in the inclusion of more extended criteria live kidney donors with excellent results.

According to current evidence and guidelines, single renal vascular anatomy is preferred in living kidney donors, although arterial or venous multiplicity (AM/VM) should not be considered as an absolute contra-indication for live kidney donation [13–20]. However, in most centers, renal vascular anatomy is one of the dominant factors in determining which kidney should be procured. In general, the kidney with the most straightforward vascular anatomy (ideally one artery, one vein) is chosen.

Recently, Fuller stated two principles regarding this issue in live donor nephrectomy [21]: First, ‘do not harm the donor’ and second; ‘make optimal use of available living donors to overcome organ shortage. It may no longer seem acceptable to exclude otherwise suitable living donors only on grounds of technical obstacles’. In light of these remarks, and since in most centers still single renal vascular anatomy is preferred [3], we decided to further study the influence of vascular multiplicity on outcome for live kidney donors and their recipients in our cohort.

We recently published a systematic review regarding extended donor criteria, including vascular multiplicity. Based on the included guidelines and available literature, it is concluded that vascular multiplicity (in particular, AM up to 3 renal arteries) should not be considered a contraindication for live kidney donation [3].

In light of this review, we decided to investigate our cohort from 2006 until 2013, including nearly 1000 living kidney donors, aiming to get a deeper insight in the shift in acceptance of donors with vascular multiplicity, their potentially increased risk for complications in the donor and the KT recipient.

## Materials and Methods

Because this study is a retrospective chart study only, analyzed anonymously, it was not considered for a full review by the Ethics Committee of the Erasmus MC, Rotterdam, The Netherlands.

No oral or written consent was retrieved for this specific chart analysis, since the number of patients is very large. The data was anonymised prior to analysis.

From January 1^st^ 2006 to December 31^st^ 2013, data of all live kidney donors and transplantations (n = 951) was collected and retrospectively reviewed. Minors receiving a live adult donor kidney were excluded (n = 37), resulting in 914 KT, including 30 patients receiving a second kidney and one patient receiving a third kidney.

In all our donors, renal vascular imaging was performed as part of a thorough standard medical screening by the nephrologist. Hereafter, each live donor is assessed by a dedicated transplant surgeon involved in live donor nephrectomy. The transplant surgeon determines which kidney will be retrieved based on the principle that the donor should be left with the best kidney and the side of the donor nephrectomy should be the least complicated one to avoid additional risk to the donor. Then, every donor is discussed prior to donation in a multidisciplinary team discussion involving (transplant) surgeons, nephrologists, anesthetists, donor coordinators, nurse practitioners and a urologist or psychologist if necessary. To discover any discrepancies in vascular anatomy between imaging and surgery, radiological and surgical reports were compared regarding vascular anatomy. The policy of our center in case of AM is that there is no contraindication for living donation, regarding the choice for left versus right kidney; the kidney with least arteries is chosen unless other factors determine that the other kidney should stay with the donor. In case of AM all transplant surgeons always aim for reconstructing the renal arteries in an end-to-side or side-to-side fashion to enable one arterial anastomosis in the recipient in order to keep the second WIT short. Only if the distance between the arteries is too large to enable safe reconstruction, separate arterial anastomoses are created in the recipient. Small (<3mm diameter) accessory arteries that are deemed not reconstructable will be ligated.

Results of the pre-operative imaging and intraoperative findings during live donor nephrectomy were correlated to several intraoperative and postoperative (surgical) outcome measures (warm ischemia time, estimated blood loss, skin-to-skin time, complications, alteration of operative technique, re-operations, length of stay, re-admission, rise in serum creatinine). Donor complications were classified according to the Clavien-Dindo classification [22].

Furthermore, intraoperative findings regarding anatomy during donor nephrectomy were correlated to several intraoperative and postoperative outcome measures of KT recipients (warm ischemia time (WIT), estimated blood loss in milliliters, skin-to-skin time, complications (scored by Clavien-Dindo), length of stay (from postoperative day one to day of discharge), 30 day re-admission rate, creatinine drop (in μmol/L, comparing preoperative values with values of the first day after surgery and with one year postoperatively), primary non-function (PNF, defined as permanent absence of graft function), delayed graft function (DGF, defined as the need for dialysis within seven days after transplantation), BPAR (defined as biopsy-proven acute rejection within three months after transplantation), diuresis of the graft on the operation table, and several other (urological) complications amongst which postoperative wound infection, wound dehiscence, urinary tract infection, urosepsis and obstruction/removal of the percutaneous nephrostomy). Since serum creatinine values can vary drastically depending on the time since last dialysis, we have chosen to include only pre-emptive recipients in the analysis of serum creatinine.

Several surgical techniques for donor nephrectomy are practiced in our center: transperitoneal laparoscopic donor nephrectomy with and without hand-assistance, hand-assisted retroperitoneoscopic donor nephrectomy and robot-assisted transperitoneal laparoscopic donor nephrectomy using the Da Vinci Surgical System. No (mini-)open donor nephrectomies have been performed since 2005.

### Statistical analysis

All analyses were conducted using IBM SPSS Statistics for Windows, Version 21.0. (IBM Corp. Released 2012. Armonk, NY: IBM Corp.) Categorical variables were compared using the Chi-square test and continuous variables were compared with the Mann-Whitney *U* test or the independent samples t-test. Graft- and patient survival were expressed using Kaplan-Meier curves, and were compared using the log-rank test. P-values less than 0.05 were considered statistically significant.

## Results

**Fig 1:**
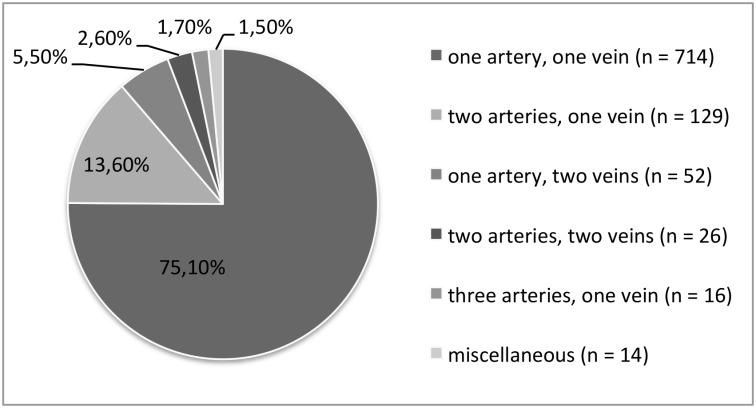
Variation in renal vascular anatomy as determined during live donor nephrectomy.

Regarding the donor baseline characteristics, of the 951 donors, 713 (75%) had single vascular anatomy of the kidney that was selected for donation. Of the other twenty-five percent, 139 (58.4%) had bilateral vascular multiplicity (Fig 1). Left-sided vascular multiplicity was present in 139 (23,5%) of left kidneys, while right-sided vascular multiplicity was present in 99 (27,6%) of right kidneys. 44,5% was male and the mean age of the donors was 52 years. In 592 (62,3%) of the donors, the left kidney was chosen. No significant differences in age, sex, Body Mass Index (BMI), choice of kidney side, ASA-classification (American Society of Anesthesiologists-classification) or surgical technique were observed between donors with single anatomy versus vascular multiplicity (Table 1). Regarding KT recipients baseline characteristics, of the 914 recipients, 64,6% was male and the mean age of the recipients was 51,3 years. No differences were found in gender, age, Body mass index (BMI), relation with the donor, preoperative serum creatinine or the percentage of pre-emptive transplantations. (Table 1)

**Table 1:** Baseline characteristics of living kidney donors and of KT recipients.

	**All live donors (n = 951)**	**Donors with single vascular anatomy (n = 713)**	**Donors with vascular multiplicity (n = 238)**	**p-value**
Gender (male; female)	423 (44,5%); 528 (55,5%)	306 (42,9%); 407 (57,1%)	117 (49,2%); 121 (50,8%);	0.093
Age (years)	52 (± 13)	52 (± 13)	51,4 (± 12,9)	0.543
ASA classification (I;II;III)	544 (61,7%); 333 (37,8%); 5 (0,6%)	410 (61,6%); 253 (38,0%); 3 (0,5%)	134 (62,0%); 80 (37,0%); 2 (0,9%)	0.706
Body Mass Index (kg/m²)	26,2 (± 3,8)	26,2 (± 3,8)	26,2 (± 3,9)	0.982
Preoperative serum creatinine (μmol/L)	74,36 (± 13,7)	74,24 (± 13,8)	74,92 (± 13,6)	0.510
Kidney (left; right)	592 (62,3%); 359 (37,7%)	45. (63,5%); 260 (36,5%)	139 (58,4%); 99 (41,6%)	0.157
**Recipient characteristics**	**All recipients (n = 914)**	**Recipients receiving kidney with single vascular anatomy (n = 688)**	**Recipients receiving kidney with multiple vascular anatomy (n = 226)**	**p-value**
Gender (male; female)	589 (64,4%); 325 (35,6%)	449 (65,3%); 239 (34,7%)	140 (61,9%); 86 (38,1%)	0.366
Age	50,9 (14,4)	51,8 (14,1)	50,2 (15,3)	0.138
BMI	25,9 (4,7)	26,1 (4,6)	25,5 (4,9)	0.109
Relation (specified direct; specified indirect; unspecified)	711 (77,8%); 136 (14,9%); 67 (7,3%)	532 (77,3%); 108 (15,7%); 48 (7,0%)	179 (79,2%); 28 (12,4%); 19 (8,4%)	0.406
Preoperative serum creatinine	643 (312)	641 (316)	652 (299)	0.654
Pre-emptive transplantation	366 (40,0%)	278 (40,4%)	88 (38,9%)	0.554

### Clinical consequences of vascular multiplicity for the donor

Donors with AM had a significantly longer warm ischemia time (WIT) (5,1 minutes versus 4,0 minutes, p = 0.016) and skin-to-skin-time (202 versus 178 minutes, p < 0.001) compared to donors with a single renal artery (SRA). No significant differences were found in estimated blood loss (EBL), complication rate, conversion to an open operative technique, re-operations, length of postoperative hospital stay, and re-admission. VM had no significant impact on WIT, EBL, conversion to an open operative technique, re-operations, length of postoperative hospital stay, or re-admissions. VM did have a significant impact on skin-to-skin time (203 minutes versus 180 minutes, p < 0.001) and complication rate (17,2% versus 8,4%, p = 0.027) (Table 2). The significance of complication rate in VM disappeared when Clavien-Dindo grade I complications (predominantly infections of the Pfannenstiel incision, hematoma formation at the incision site (opened at the bedside), and urinary retention not requiring insertion of a urinary catheter) were removed. Thus, considering complications ranked grade II and higher, no significant differences are found (p = 0.617).

**Table 2:** Intra- and postoperative outcome for donors with single renal arterial and venous anatomy compared to donors with multiple renal arteries and veins.

	**Donors with single renal arterial anatomy (n = 771)**	**Donors with multiple renal arteries (n = 180)**	**p-value**
Warm ischemia time (minutes)	4,0 (2,2)	5,1 (5,7)	**0.016**
Estimated blood loss (ml)	151 (226)	207 (416)	0.099
Skin-to-skin time (minutes)	178 (48)	202 (45)	**<0.001**
Complication rate (Total number of complications according to the Clavien-Dindo classification)	68 (8,8%)	20 (10,1%)	0.339
Conversion to open technique	7 (0,9%)	3 (1,7%)	0.482
Re-operation	6 (0,8%)	2 (1,1%)	0.660
Postoperative length of stay (days)	3,44 (1,42)	3,50 (1,54)	0.639
Re-admission	12 (1,6%)	4 (2,2%)	0.532
Difference in serum creatinine^a^ (μmol/L)	41,4 (12,8)	40,6 (13,0)	0.457
Difference in serum creatinine^a^ (%)	56,3% (16,3)	55,2% (17,3)	0.424
Difference in serum creatinine^b^ (μmol/L)	34,6 (12,2)	35,7 (13,4)	0.334
Difference in serum creatinine^b^ (%)	47,3% (15,7)	49,5% (18,5)	0.172
	**Donors with single renal venous anatomy (n = 862)**	**Donors with multiple renal veins (n = 89)**	**p-value**
Warm ischemia time (minutes)	4,2 (3,3)	4,6 (2,8)	0.240
Estimated blood loss (ml)	161 (271)	168 (296)	0.802
Skin-to-skin time (minutes)	180 (47)	203 (50)	**<0.001**
Complication rate (Total number of complications according to the Clavien-Dindo classification)	72 (8,4%)	16 (17,2%)	**0.027**
Conversion to open technique	8 (0,9%)	2 (2,2%)	0.312
Re-operation	7 (0,8%)	1 (1,1%)	0.795
Postoperative length of stay (days)	3,44 (1,42)	3,60 (1,60)	0.289
Re-admission	14 (1,6%)	2 (2,2%)	0.712
Difference in serum creatinine^a^ (μmol/L)	41,3 (12,8)	40,1 (13,3)	0.405
Difference in serum creatinine^a^ (%)	56,4% (16,5)	53,4% (15,9)	0.105
Difference in serum creatinine^b^ (μmol/L)	34,8 (12,5)	34,4 (11,8)	0.798
Difference in serum creatinine^b^(%)	47,9% (16,3)	46,1% (15,3)	0.395

Continuous data represented as mean (sd)

^a^: difference of serum creatinine between the first day after surgery and preoperative values

^b^: difference of serum creatinine between year one postoperative and preoperative values

In 32 (3,4%) cases, there was an intraoperative change in surgical technique. In 25 (2,7%) cases, single anatomy was present, and in 7 (0,6%) cases, the donor had vascular multiplicity. No significant differences were found. (p = 0.614). In 10 (1%) cases, surgery was converted to open surgery, and in the remaining cases, conversion was done from laparoscopic transperitoneal to hand-assisted transperitoneal, HARP to laparoscopic, robot-assisted to HARP or laparoscopic and robot-assisted continued with a handport. No significant differences were observed between single anatomy and vascular multiplicity (p = 0.186 for conversion to open technique, p = 0.614 for alteration in surgical technique). When scored according to the Clavien-Dindo classification, overall complications occurred as shown in Table A in S1 Data. One donor with a history of myocardial infarction developed ventricular bradycardia immediately postoperatively, had to be resuscitated but unfortunately did not survive.

### Clinical consequences of vascular multiplicity for the KT recipient

No significant differences were found in patient survival (p = 0.148 for AM-donors and p = 0.101 for VM-donors) and graft survival (p = 0.610 for AM-donors and p = 0.573 for VM-donors) (Figs A, B, C and D in S1 Data, median follow-up of 50 months). Patients receiving a kidney with AM had a significantly longer second WIT (25 minutes versus 23 minutes, p = 0.008) and total warm ischemia time (30 versus 27 minutes, p = 0.002). No significant differences were noted in skin-to-skin time, EBL, postoperative length of stay or 30-day readmission rate. Regarding venous anatomy, no significant differences were found in above-mentioned parameters when comparing single and multiple anatomy. (Table 3)

**Table 3:** Intra- and postoperative outcome of patients receiving kidneys with either single or multiple arterial or venous anatomy.

	**Single arterial anatomy (n = 740)**	**Multiple arterial anatomy (n = 174)**	**p-value**
2^nd^ WIT (minutes)	22,75 (8,02)	24,98 (10,10)	**0.008**
Total warm ischemia time (minutes)	26,73 (8,51)	30,02 (12,91)	**0.002**
Estimated blood loss (ml)	403,02 (489,72)	453,26 (706,00)	0.396
Skin to skin time (minutes)	132 (36)	135 (32)	0.515
Creatinine drop (μmol/L)^a^	291 (45%)	279 (40%)	0.666
Creatinine drop (μmol/L) ^b^	489 (74%)	515 (74%)	0.606
Postoperative length of stay (days)	14,47 (10,870)	14,49 (9,276)	0.983
Readmission within 30 days	134 (19,5%)	39 (17,3%)	0.454
	**Single venous anatomy (n = 833)**	**Multiple venous anatomy (n = 81)**	**p-value**
2^nd^ WIT (minutes)	23,08 (8,565)	24,15 (7,722)	0.283
Total warm ischemia time (minutes)	27,22 (9,75)	28,93 (8,14)	0.133
Estimated blood loss (ml)	416,52 (551,083)	372,60 (374,579)	0.506
Skin-to-skin time (minutes)	133 (35)	136 (39)	0.562
Creatinine drop (μmol/L)^a^	290 (45%)	261 (41%)	0.466
Creatinine drop (μmol/L)^b^	493 (74%)	483 (75%)	0.155
Postoperative length of stay (days)	14,52 (10,863)	14,04 (7,102)	0.698
Readmission within 30 days	59 (8,6%)	22 (9,7%)	0.599

^a^: difference of serum creatinine in pre-emptive KT recipients between the first day after surgery and preoperative values

^b^: difference of serum creatinine in pre-emptive KT recipients between year one postoperative and preoperative values

No significant differences were found in occurrence of PNF, diuresis of the transplanted kidney on the operation table before wound closure, thrombosis rate or urologic complications (defined as: necessitating a percutaneous nephrostomy) when comparing single arterial anatomy and AM. The rate of DGF was significantly increased in non-preemptive renal transplant recipients receiving a kidney with AM (p = 0.001). Interestingly, recipients of a kidney with single arterial anatomy had a significantly higher rate of BPAR (p = 0.012). This is an interesting finding that we cannot explain as the mean number of HLA-mismatches was exactly the same in the whole group (a mean of 3,3 HLA-mismatches, p = 0.885) and no significant differences were seen between mean HLA-mismatches in the BPAR-group comparing AM with a single artery (p = 0.511, data not shown). Moreover, the percentage of retransplantations in recipients suffering from BPAR in the single arterial anatomy group was not significantly higher (p = 0.945, data not shown). No significant differences were found in occurrence of diuresis before wound closure, thrombosis rate, PNF rate, PCN-insertion rate or rejection rate when comparing single venous anatomy and VM. Regarding complication rate in the recipients, we examined: postoperative wound infection, wound dehiscence, urinary tract infection, urosepsis and splint obstruction/removal. No significant differences were found in incidence of these complications when comparing single and multiple arterial and venous anatomy (Table 4). If an accessory lower pole artery was present, it did not lead to an increased number of inserted percutaneous nephrostomies (p = 0.409)

**Table 4:** Postoperative outcome of kidney transplant patients comparing single and multiple arterial or venous anatomy.

	**Single arterial anatomy (n = 740)**	**Multiple arterial anatomy (n = 174)**	**p-value**
Diuresis directly after reperfusion (yes; minimal)	643 (91,7%); 10 (1,4%)	48 (6,8%); 1 (0,6%)	0.640
Thrombosis	9 (1,2%)	4 (2,3%)	0.281
PCN	110 (15,0%)	22 (12,7%)	0.446
PNF	4 (0,5%)	2 (1,1%)	0.371
DGF^1^	30 (6,9%) (n = 437)	15 (13,9%) (n = 108)	**0.018**
BPAR^2^	103 (13,9%)	12 (6,9%)	**0.012**
Mean number of HLA-mismatches	3.3	3.3	0.885
Retransplantations	25 (3,4%)	6 (3,4%)	0.963
Wound infection	18 (2,4%)	5 (2,9%)	0.749
Wound dehiscence	7 (0,9%)	0 (0,0%)	0.196
Splint obstruction/removal	27 (3,6%)	12 (6,7%)	0.063
Urinary tract infection	125 (16,9%)	31 (17,7%)	0.800
Urosepsis	13 (1,8%)	2 (1,1%)	0.564
	**Single venous anatomy (n = 833)**	**Multiple venous anatomy (n = 81)**	**p-value**
Diuresis directly after reperfusion (yes; minimal)	729 (91,9%); 10 (1,3%)	68 (89,5%); 1 (1,3%)	0.735
Thrombosis	11 (1,3%)	2 (2,5%)	0.401
PCN	121 (14,6%)	11 (13,8%)	0.831
PNF	6 (0,7%)	0 (0,0%)	0.443
DGF^1^	39 (7,9%) (n = 494)	6 (11,8%) (n = 51)	0.339
BPAR^2^	105 (12,6%)	10 (12.3%)	0.946
Mean number of HLA-mismatches	3.3	3.1	0.07
Retransplantations	26 (3,1%)	5 (6,2%)	0.147
Wound infection	22 (2,6%)	1 (1,2%)	0.424
Wound dehiscence	6 (0,7%)	1 (1,2%)	0.630
Splint obstruction/removal	38 (4,5%)	1 (1,1%)	0.122
Urinary tract infection	141 (17,0%)	15 (18,1%)	0.799
Urosepsis	15 (1,8%)	0 (0,0%)	0.217

^1^. Analysis of DGF excluding pre-emptive transplantations.

^2^. Biopsy-proven acute rejection within 3 months after transplantation.

### Limitations

First, this is a retrospective study design with all the limitations inherent to the lack of prospective follow-up, and we recognize the possibility of information bias. Furthermore, by including many outcome parameters, statistically one in twenty should turn out significant.

Second, renal vascular anatomy may be misclassified since radiologists and transplant surgeons each might have a different way of judging anatomy. For example, a transplant surgeon might have a more functional look at the present anatomy. Early branching of a singular renal artery, thus requiring separate dissection and clamping of both arterial branches, might be classified as two renal arteries. This example might have been correctly seen by the radiologist as a single renal artery with early branching and noted as such. This example may lead to an incorrect false negative imaging result. To correct for this possibility, all false negative imaging results have been examined independently (JAL/MB). In case of doubt, cases were reviewed by a supervising author (FJMFD). The reverse is also possible, however. Early branching may be misdiagnosed by a radiologist as dual arteries [23].

Third, we do not have any data of the sizes of the multiple vessels and their distances apart. For example, as two moderately sized arteries in very close proximity could be handled differently than two arteries, of which one is a very small lower pole artery that is separated by several centimeters from the dominant upper pole artery.

Fourth, considering the low number of donors presenting with rare anatomical variations (i.e. more than 3 arteries or veins, 3,1% total), no reliable conclusion can be made regarding the feasibility and safety of kidney donation in these cases. In our cohort, only 22 kidneys (2,3%) had three renal arteries and only one kidney (0,1%) had four arteries. Similarly, five kidneys (0,5%) had three veins, three (0,3%) had four veins and only one had five veins (0.1%) (Fig 1). Moreover, these donors were carefully selected, and we did not analyze the potential donors with vascular multiplicity that were excluded, early in the screening process. Even in a high volume center as ours, with almost 1000 live donor nephrectomies performed in eight years, even higher numbers would be needed to get a sufficient insight in these rare anatomical variations. It would therefore seem prudent to create an international database containing information about these cases. Only in this way, can we collect enough information on the practice of live kidney donation to reliably assess potential donors presenting with these anatomical variations.

Last, we were not able to analyze the number of vascular reconstructions ‘on the bench’, because of the relatively scarce reporting of these interventions in the operation reports. In addition, the exact type of reconstruction made (e.g. side-to-side or end-to-side anastomosis, becoming increasingly complex with an increasing number of arteries or veins) was often unclear. One could imagine that the need for a reconstruction could possible lead to a higher incidence of complications in a recipient. Furthermore, if a reconstruction was performed, the skin-to-skin time of the donor is longer, thereby introducing a confounder in the analysis of the donor outcome.

As stated in the introduction, only one guideline incorporated vascular multiplicity of possible live kidney donors (the British Transplantation Society) [16]. Although several studies have looked into vascular multiplicity, still, no hard statements are written regarding this issue. Some studies show an increased incidence of (ureteral) complications in recipients [10,24], whilst other show good outcome [11,25–28].

## Discussion

This study provides a meticulous overview and analysis of data of a large cohort of live kidney donors over a period of 8 years. In this time-period, only laparoscopic donor nephrectomies or other endoscopic techniques were performed, diminishing possible confounding. The findings of this study prove that renal vascular multiplicity in a living kidney donor should not be considered as a contraindication for kidney donation, provided an adequate preoperative workup has been performed, and the surgical team is experienced. WIT and skin-to-skin time are both significantly longer in donors with AM, and VM was only associated with a significantly longer skin-to-skin-time. In the VM group, the WIT was not longer, what can be explained by the fact that often smaller accessory veins are ligated before ligating the renal artery, after which the WIT starts. However, the impact of AM/VM on clinical outcome is small to non-existent. Furthermore, we have found no significant difference in parameters that would impact a donors’ outcome, such as alteration of operative technique, conversion to open technique, length of stay, likelihood of reoperation or need for re-admittance. Although the complication rate was significantly higher for VM, this was only the case for Clavien-Dindo grade I complications. It has been postulated that the presence of lower pole arteries leads to more urological complications because of the fact that they normally vascularize the ureter. In contrast to the findings of Kok et al[12]., reporting a significantly increased risk of ureteral complications in patients with an accessory lower pole artery, in our cohort the presence of additional lower pole arteries did not lead to an increased number of inserted percutaneous nephrostomies.

Looking at parameters related to recipient outcome, we found that AM/VM does not lead to increased morbidity and mortality. We also found no significant differences in intraoperative parameters such as EBL or skin-to-skin-time in KT. Second WIT was significantly longer for kidneys with multiple renal arteries, however, not leading to impaired outcome for the recipient.

Recently, Omoto et al. have analyzed their cohort of 533 live kidney donors [26], looking only at the number of renal arteries, showing that AM might increase slow graft function (defined as: serum creatinine level is more than 3.0 mg/dL at 4 days after transplantation) but not acute rejection rates. In our cohort, we have also analyzed the presence of multiple veins. In general, the incidence of slow (SGF) and DGF is low in grafts procured from live kidney donors. The total rate of DGF in our cohort was 5,1% (n = 47). The incidence of DGF was significantly increased when transplanting a kidney with vascular multiplicity. One can argue whether these outcome measures are clinically relevant to analyze in grafts from live kidney donors, even if vascular multiplicity is present, because of the low incidence of DGF in kidneys from living donors. Furthermore, in our centre, a large percentage (40,7%) of the donations is done for preemptive transplantation. In our cohort, the mean WIT is increased by only two minutes due to VM. More importantly, graft- and patient survival are not negatively influenced by vascular multiplicity.

By performing this analysis of our cohort, consisting of a large number of live kidney donors and recipients, in conclusion, we have shown that live donor nephrectomy is a safe procedure for the donor, even with vascular multiplicity. Although vascular multiplicity is considered as a relative contra-indication to donation in current guidelines, based on our results we conclude that it should not be considered a contraindication at all. Obviously, robust screening is warranted in potential live kidney donors to ensure donor safety. Furthermore, in contrast to published literature [5,12,21] KT recipients receiving a kidney from a live donor with vascular multiplicity have excellent outcome as well, and no increased risk for urological complications.

## Supporting Information

S1 Data Surgical techniques used for live donor nephrectomy. Fig A: Patient survival regarding arterial anatomy. Fig B: Graft survival regarding arterial anatomy. Fig C: Patient survival regarding venous anatomy. Fig D: Graft survival regarding venous anatomy. Fig E: Proportion of imaging techniques over the years. Fig F: Chosen side (left versus right) of the procured kidney. Table A: Overall complications when score according to the Clavien-Dindo classification. Table B: Conversion rates to the open technique. Table C: Conversion rates to other surgical techniques.
(DOCX)

## Abbreviations

MRA: Magnetic Resonance Angiography
CTA: Computed Tomography Angiography
ESRD: end stage renal disease
AM: arterial multiplicity
VM: venous multiplicity
DSA: digital subtraction angiography
ASA-classification: American Society of Anesthesiologists-classification
BMI: body mass index
WIT: warm ischemia time
SRA: single renal artery
SRV: single renal vein
EBL: estimated blood loss
PCN: percutaneous nephrostomy
KT: kidney transplant
DGF: delayed graft function
PNF: primary non-function
BPAR: biopsy-proven acute rejection
