# Supplementary material for: Vascular Multiplicity Should Not Be a Contra-Indication for Live Kidney Donation and Transplantation
Source: PLoS One. 2016 Apr 14;11(4):e0153460. doi: 10.1371/journal.pone.0153460 (PMC4831799; doi:10.1371/journal.pone.0153460)
Supplement: S1 Data — Fig A: Patient survival regarding arterial anatomy. Fig B: Graft survival regarding arterial anatomy. Fig C: Patient survival regarding venous anatomy. Fig D: Graft survival regarding venous anatomy. Fig E: Proportion of imaging techniques over the years. Fig F: Chosen side (left versus right) of the procured kidney. Table A: Overall complications when score according to the Clavien-Dindo classification. Table B: Conversion rates to the open technique. Table C: Conversion rates to other surgical techniques. (DOCX) [file pone.0153460.s001.docx]

**SUPPLEMENTAL DATA**

*Surgical techniques [*[*1*](#_ENREF_1)*];*

**Laparoscopic donor nephrectomy:** First, the donor is positioned in right or left lateral decubitus position. Then, the first trocar is inserted periumbilically and a pneumoperitoneum is created by CO2 insufflation, after which a 30° video-endoscope is introduced and depending on the side three to four additional trocars are inserted. Depending on a right or left-sided nephrectomy, the right or left hemicolon is dissected from the lateral abdominal wall and mobilized medially. Gerota’s fascia is opened and the kidney is exposed from a varying amount of surrounding perirenal fat. Next, the ureter is exposed until it crosses the gonadal vein. The renal vessels are dissected and encircled with red or blue vessel loops to facilitate identification of the artery and vein from different directions, respectively, and allow for safe manipulation of the vessels. The venous branches of the renal vein, especially in case of left sided donor nephrectomy, are clipped and divided with scissors. When the kidney, ureter, vein and artery are all fully dissected, a 5 to 8 cm horizontal suprapubic incision or Pfannenstiel incision is made as extraction site. An endobag is introduced via a small incision in the peritoneum. Subsequently, the distal ureter is clipped and divided with scissors, secondly the renal artery is divided with an endostapler and lastly, the renal vein is divided with an endostapler. The kidney is placed in the endobag and extracted via the incision.

**Hand-assisted retroperitoneoscopic donor nephrectomy:** Using this technique, a 7-10 cm Pfannenstiel incision is made, through which, after blunt dissection to create a retroperitoneal space, a hand port is inserted. Blunt introduction of the first trocar between the iliac crest and the hand port is guided by the surgeon’s hand inside the abdomen. CO2 is insufflated retroperitoneally to 12 cm H2O pressure. Two other 10-12 mm trocars, respectively just outside the midline inferior to the costal margin and in the flank, are inserted to create a triangular shape. Dissection of the kidney and renal vessels is similar to transperitoneal donor nephrectomy but with hand-assistance and from a slightly different angle. The kidney is removed manually. Two advantages of this technique are avoiding the peritoneal cavity and hand-assistance while operating.

**Robot-assisted laparoscopic donor nephrectomy:** In this technique, the operating surgeon sits behind a console operating with three robot-arms while the operating assistant stands beside the patient. The position of the trocars and extraction site of the kidney is comparable to conventional laparoscopic donor nephrectomy. The advantages of this technique are the three-dimensional magnified vision, 560 degrees rotation capability, the comfort for the surgeon, and elimination of tremor.

In case of donor AM, we routinely performed an end-to-side or side-to-side reconstruction ‘on the bench’ straight after the kidney extraction during donor nephrectomy to minimize the number of anastomoses to be made during implantation and the associated longer warm ischemia time during transplantation. If these reconstructions were not feasible, multiple arterial anastomoses were made.

In case of multiple veins, we normally choose to sacrifice all but the dominant vein, since there should be .

**Implantation:** A semi‐lunar supra‐inguinal incision is made in the left or right lower quadrant of the abdomen. The fascia, external oblique, transversus abdominus, and internal oblique muscle are divided. The inferior epigastric vessels are divided to gain access to the preperitoneal space. In males, the spermatic cord evidently should be preserved, but in women the round ligament of the uterus is divided. When the iliac fossa is exposed and the external iliac artery and vein are prepared, the venous anastomosis (end‐to‐side) is created first. After creating the arterial end‐to‐side anastomosis, the perfusion is restored. After reperfusion, the ureter‐vesical anastomosis is created with an extra‐vesical anastomosis by Lich‐Gregoir [[2](#_ENREF_2)] or an intra-vesical anastomosis by Politano-Leadbetter [[3](#_ENREF_3)]. Before closure of the fascia, muscles and skin, a percutaneous drain is placed.

**Supplemental Figures**


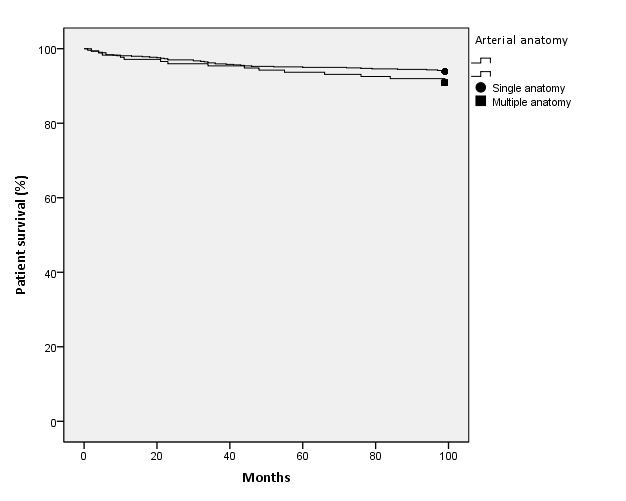


Supplemental figure A.


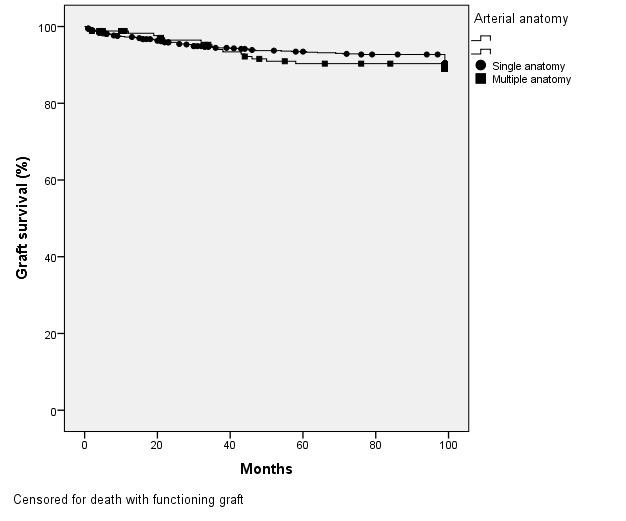


Supplemental figure B.


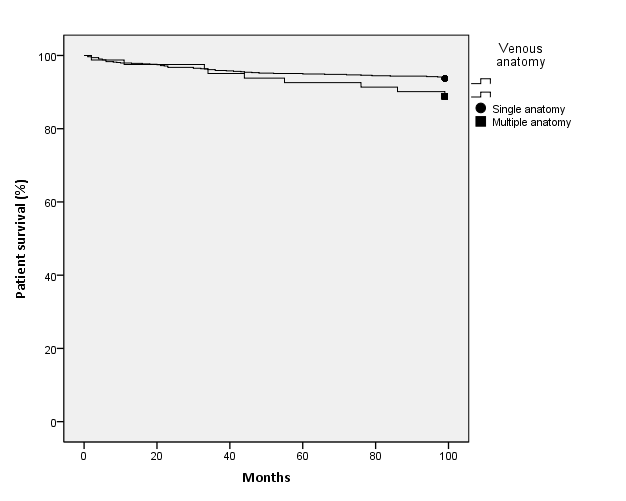

Supplemental figure C.


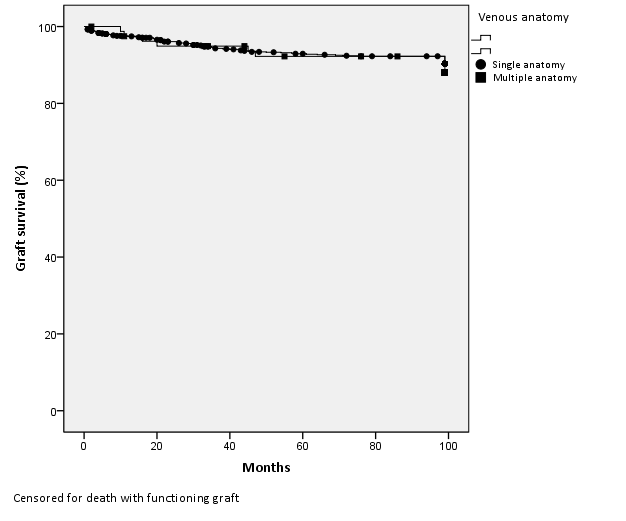


Supplemental figure D.

Supplemental figure E.

Supplemental figure F.

| **Grade** | **Percentage (n)** | **Percentage in donors with single anatomy (n)** | **Percentage in donors with multiple anatomy (n)** |
| --- | --- | --- | --- |
| No complications | 90,7% (863) | 91,7% (654) | 87,8% (209) |
| Grade I | 4,5% (43) | 3,9% (28) | 6,3% (15) |
| Grade II | 3,5% (33) | 3,2% (23) | 4,2% (10) |
| Grade IIIa | 0,1% (1) | 0,1% (1) | 0% |
| Grade IIIb | 0,8% (8) | 0,7% (5) | 1,3% (3) |
| Grade IVa | 0,2% (2) | 0,3% (2) | 0% |
| Grade IVb | 0% | 0% | 0% |
| Grade V | 0,1% (1) | 0% | 1 (0,4%) |

Supplemental Table A.

|  | **Single anatomy** | **Vascular multiplicity** | **p-value** |
| --- | --- | --- | --- |
| Total conversion rate (conversion to open technique) | 5 (0,7%) | 5 (2,1%) | 0.186 |
| Laparoscopic transperitoneal to open | 4 (0,6%) | 4 (1,7) |  |
| Hand-assisted retroperitoneoscopic to open | 1 (0,1%) | 1 (0,4%) |  |

Supplemental table B.

|  | **Single anatomy** | **Vascular multiplicity** | **p-value** |
| --- | --- | --- | --- |
| Total alteration rate | 25 (3,5%) | 6 (3,0%) | 0.614 |
| Laparoscopic transperitoneal to hand-assisted transperitoneal | 12 (1,7%) | 6 (2,5%) |  |
| Hand-assisted retroperitoneoscopic to laparoscopic transperitoneal | 5 (0,7%) | 1 (0,5%) |  |
| Robot-assisted to hand-assisted laparoscopic | 2 (0,3%) | 0 |  |
| Robot-assisted to transperitoneal laparoscopic | 1 (0,1%) | 0 |  |
| Robot-assisted continued with handport | 5 (0,7%) | 0 |  |

Supplemental table C.

1. Janki, S., F.J.M.F. Dor, and J.N.M. Ijzermans, *Surgical aspects of live kidney donation: an updated review.* Front Biosci (Elite Ed), 2015. **7**: p. 394-416.

2. Gregoir, W., *[THE SURGICAL TREATMENT OF CONGENITAL VESICO-URETERAL REFLUX].* Acta Chir Belg, 1964. **63**: p. 431-9.

3. Politano, V.A. and W.F. Leadbetter, *An operative technique for the correction of vesicoureteral reflux.* J Urol, 1958. **79**(6): p. 932-41.
